# L-Box Form Filling of Thixotropic Cementitious Paste and Mortar

**DOI:** 10.3390/ma13071760

**Published:** 2020-04-09

**Authors:** Mareike Thiedeitz, Nasime Habib, Thomas Kränkel, Christoph Gehlen

**Affiliations:** Centre for building materials, Technical University of Munich, 81245 Munich, Germany; nasime.h@hotmail.com (N.H.); thomas.kraenkel@tum.de (T.K.); gehlen@tum.de (C.G.)

**Keywords:** rheology, form-filling ability, thixotropy, workability

## Abstract

Rheological properties of cementitious pastes and mortar affect the casting, placement, and setting properties of fresh concrete. Fundamental rheological knowledge thus helps in predicting concrete flowability and workability. Empirical equations correlate actual rheological parameters based on physical material characteristics to workability tests. Still, these equations generally only take the dynamic yield stress of the material into account. This is not sufficient for thixotropic cementitious pastes or mortars, which possess structural buildup at rest. Workability predictions regarding the flow of concrete are thus more complicated with thixotropic materials. During form-filling in L-shaped formworks, the flow velocity of concrete slows down, wherefore rheological parameters change with time. At initial fast flow, high shear rates without structural buildup can be assumed. Dynamic yield stress and a steady state viscosity thus are proper parameters for empirical equations describing concrete flowability. During low shear rates, partial structural buildup takes place. Viscosity and yield stress increase due to agglomeration and affect the flowability of concrete tremendously. Rheological parameters of various cementitious pastes and mortars varying in their solid volume fraction and flowability were investigated in a vane-in-cup rheometer. The workability of the same mixtures was investigated by measuring the flow length in an L-shaped formwork. The effect of yield stress, viscosity, and thixotropic structural buildup on flow length was investigated. Subsequently correlations and discrepancies between flowability parameters and workability equations were analyzed.

## 1. Introduction

Concrete is a non-Newtonian material with yield stress and viscosity ranging from shear-thinning to shear-thickening due to its mixture and design, e.g., its relative solid volume fraction and the chemistry of the cementitious phase [1,2,3,4]. Knowledge about fresh concrete flow is essential for casting, placement, and setting processes which affect the strength and durability performance of hardened concrete [5,6,7,8,9]. In particular, the casting of densely reinforced or complex construction elements requires a deep understanding of the rheological behavior of concrete. The rheological behavior is expressed by the parameters yield stress, viscosity, and structural buildup, which are investigated by conducting rheometric measurements. Rheometric devices nevertheless are most often expensive and not applicable at construction sites. Therefore, flowability tests are conducted for the estimation of workability properties. These methods are known as empirical stoppage tests (e.g., those developed in [10,11]) and are also used in standardizations [12]. Empirical equations correlate rheological parameters with actual workability properties. Still, with more complex material properties or placement requirements there is a discrepancy between rheometry data and empirical stoppage tests. Densely packed cementitious suspensions possess slow flow velocities; hence, the rheological properties are highly time- and hydration-dependent. The regression of flow behavior on Bingham parameters calculated from steady shear velocities is not sufficient for the prediction of real flow. In particular, at rest or slow flow, yield stress increases due to structural buildup, which is the sum of irreversible chemical nucleation hydration and reversible colloidal agglomeration forces [13,14,15,16,17]. Moreover, the rheological parameters of yield stress and viscosity are both dependent on the current shear rate [15,18,19]. Still, empirical correlations most often consider concrete as a Bingham-like material. Numerical analysis and simulation approaches generally implement Bingham parameters as well. For standard concretes with low structural buildup or self-compacting concrete (SCC) with fluid-like behavior, these simulations are viable [20,21]. Indeed, for concretes that contain high amounts of colloidal cementitious paste, many admixtures, and low water-to-cement ratios, the input parameters for simulations need to be further investigated. Thus, in this paper the results of an empirical stoppage test with an L-shaped formwork for construction site applications are compared with investigations of complex rheological behavior using a highly sensitive rheometer. A stepwise comparison approach between rheological parameters and the real flow showed the loss of current fitting equations. The interdependencies between the rheological parameters were shown. Subsequently, rheological and workability data were combined for a more enhanced flow prediction of concrete. For this reason, cement pastes and mortars with two different relative solid volume fractions of the cementitious paste were investigated. For each mixture with different solid volume fractions, the flowability was varied by the dosage of superplasticizer. Resulting yield stress, viscosity, and thixotropy values were calculated and compared to the workability values from form-filling values in the L-shaped formwork.

## 2. Rheological Background

### 2.1. Rheological Parameters Due to Colloidal Particle Structuration

Generally, concrete starts to flow as soon as induced shear stress overcomes its yield stress. Yield stress is defined by the particle structural network which is dependent on packing density and relative solid volume fraction, colloidal interparticle forces, and the tendency for agglomeration [22,23,24,25,26,27]. Due to particle interactions, colloidal and non-colloidal cement particles of up to 100 µm tend towards agglomeration. The sum of attractive and repulsive forces defines agglomeration kinetics [28]. Attractive forces include Van der Waals forces, electrostatic attraction, and Brownian motion. Repulsive forces include electrostatic repulsion and steric hindrance. Viscosity is the flow velocity or the resistance against shear of a concrete once flow occurs. It is affected mostly by frictional forces which occur between the particles in the suspension [29,30,31]. Therefore, the apparent viscosity increases with the relative solid volume fraction of particles $\Phi_{rel}$ (ratio of the solid concentration to the maximum solid concentration $\Phi /\Phi_{max}$). With increasing $\Phi_{rel}$, the distance between the particles in the suspension decreases, wherefore the strength of the particle network increases [32]. 

Structural buildup is the sum of reversible structural buildup, called thixotropy, and chemical hydration nucleation, which first leads to ettringite and subsequently to irreversible C-S-H bridging of the reactive cementitious phase [13]. The tendency for agglomeration and subsequent deagglomeration (when shear is applied) is also dependent on the strength of the particle network. Reversible structural buildup takes place during the first minutes of rest or slow flow, whereas structural breakdown occurs within a few seconds once shear occurs [6,16]. Irreversible structural buildup due to the hydration process takes place as soon as water is added to the reactive mixture. The speed of hydration as well as the evolution of hydration products meanwhile is dependent on the chemical composition of the cement, additives, and admixtures. In summary, the actual rheological parameters evolve with shear and time [18,33,34].

(1) $$\tau_{0}\left(t\right)=\tau_{0,d}+A_{thix}\times t$$

(2) $$\eta \approx \;\eta_{0}\left(1+\lambda^{n}\right);\;\frac{d\lambda }{dt}=\frac{1}{\Theta }-\alpha \dot{\gamma \lambda }$$

**Figure 1:**
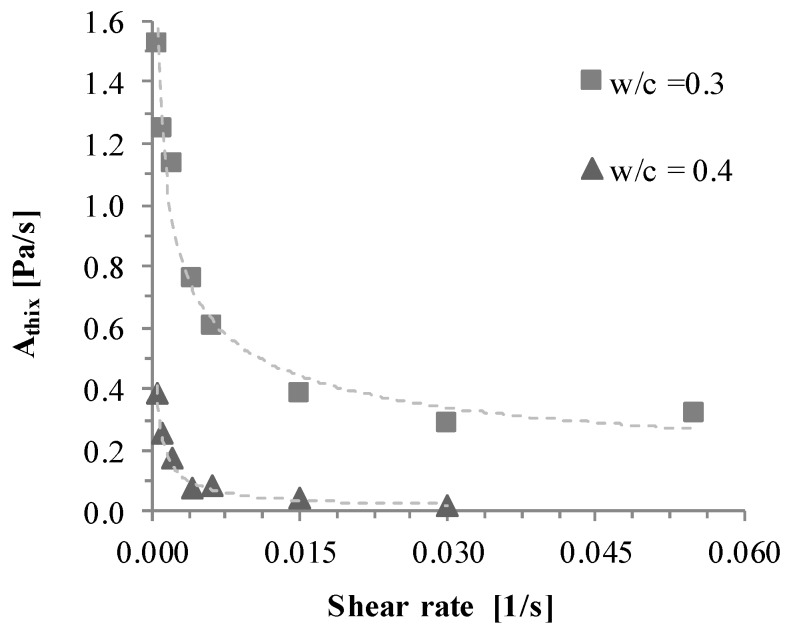
Residual structural buildup as a function of shear rate [36]. w/c: water-to-cement ratio.

In Equation (1), the yield stress as a function of time $\tau_{0}\left(t\right)$ is expressed as static yield stress $\tau_{0,d}$, which evolves due to the thixotropy parameter $A_{thix}$. The thixotropy parameter $A_{thix}$ in Pa/s was invented as the slope of yield stress increase measured in a static yield stress test [16,19]. The parameter $A_{thix}$ meanwhile only constitutes the yield stress increase during rest. As has already been reported [15,16,18,34], the real structural buildup is dependent on the apparent shear. Therefore, during low shear, only a percentage of $A_{thix}$ should be considered for the evolution of static yield stress, as was also shown in investigations in [35] (compare Figure 1). The residual $A_{thix}$ was investigated depending on the applied low shear during “time at rest” for series of cement pastes with water-to-cement ratios (w/c) of 0.3 and 0.4, respectively. 

Equation (2), invented by Coussot et al. [33], presents the dependence of viscosity η on the structural parameter λ with the power of n, which represents the ratio between structural buildup and breakdown. With derivation of time and depending on an initial viscosity $\eta_{0}$, λ increases depending on the flocculation time Θ, the structural parameter α, and the shear rate $\dot{\gamma }$. In summary, it can be stated that during steady shear and a high shear rate for full structural breakdown, rheological parameters remain nearly constant and are predictable when there is knowledge on the degree of agglomeration. As soon as the shear gets too low for full deagglomeration of the particle network, the values of the rheological parameters yield stress and viscosity increase. 

### 2.2. Rheological Measurements 

Rheological parameters obtained from rheometric investigations have different drawbacks. They are not only shear rate-dependent but are highly sensitive to incorrect measurements [36] and rely on various factors, e.g., sample preparation or the method of rheological investigation. Depending on shear history, rheological parameters are able to vary by more than 30% [37]. Even with the same sample preparation, the choice of the rheometric device and its geometry highly affects the outcome of rheological investigation, as was shown by recently conducted round-robin tests on cement and concrete rheology [38]. Moreover, the calculation from measured torque to shear stress as well as the regression function for the calculation of rheological parameters has an impact on the final rheological parameters. Rheometers with vane-in-cup devices measure the torque within the suspension. The actual shear stress can be calculated depending on the device’s geometry and the sheared part of the suspension, which is hardly measurable. The sheared part of the suspension also is a function of its actual rheological parameters and the rotational speed. Thus, rheological parameters from vane-in-cup rheometers are solved iteratively. Inaccuracy increases as the tested sample leaves the Newtonian region. Absolute parameters using parallel-plate or cone-plate geometries provide reliable data on rheological properties, although they are only available for suspensions without coarse aggregates [39]. With cement as reactive phase and the associated structural buildup taking place as soon as the sample is at rest, the overall procedure and time steps affect the final rheological measurement. 

### 2.3. Empirical Stoppage Tests as Rheological Measuring Tool

Rheological parameters state the physical material response to outer shear and need to be known for a fundamental understanding of flow behavior. A proper prediction of concrete flowability is of practical interest, not only for the knowledge of the material’s natural flow manner but also for workability characteristics which ensure correct placement and setting properties [20,21,40]. Still, flowability described by rheological parameters and workability are rarely investigated similarly. The investigation of rheometric parameters provides information regarding the “inner” flow characteristics and links rheometric values to actual physical and chemical characteristics of the material [41,42], whereas empirical stoppage tests provide information regarding placement and setting properties, namely “external” flow characteristics. A correlation between rheological parameters from highly sensitive rheometers and workability properties is of practical interest for enhanced placement properties. Each measurement technique has its benefits. While rheological parameters assume knowledge about absolute material behavior (despite uncertainties, see Section 2.2), workability tests are not related to *real* physical properties but give an impression about placement and setting properties. 

Highly sensitive rheometers that provide comprehensive rheological information are most often not applicable for construction sites. Therefore, empirical equations assume a correlation between rheology and workability. Common so-called empirical stoppage tests are e.g., the mini slump flow test and the L-box test. Roussel et al. assume a correlation between the mini slump flow value (according to EN 1015-3:2007-05 [43] and EN 12350-8:2010-12 [44]) and the yield stress [10] (see Equation (3)), with ρ being the density in (kg/m^3^), g the gravity = 9.81 m/(s^2^),V the volume of the mini slump cone in (m^3^), and R the measured mini slump flow radius in (m).

(3) $$\tau_{0}=\frac{225\rho gV^{2}}{128\pi^{2}R^{5}}$$

The L-box test (according to EN 12530-10 [12]) or tests with the LCPC-box [45] also correlate a flow length of concrete to the yield stress, which is shown in Equation (4) from Nguyen et al. [11], where the flow length L in m is correlated to the geometric conditions of the flow box ($l_{0}$ being the width of the flow box and $h_{0}$ the height of concrete after flow at the beginning of the channel, both in (m)) and the suspensions’ yield stress $\tau_{0}$ in (Pa)

(4) $$L\;=\frac{h_{0}\rho gl_{0}}{2\tau_{0}}+\frac{l_{0}^{2}\rho g}{4\tau_{0}}\ln\left(\frac{l_{0}}{l_{0}+2h_{0}}\right)$$

Therefore, with a rheometric result of a flow curve and subsequent regression of the yield stress $\tau_{0}$, it is assumed that concrete flow or workability is predictable. Various researchers correlated L-box flow of flowable or self-compacting concrete to Bingham parameters [46,47]. Bingham parameters are often used as input parameters for computational fluid dynamics (CFD) simulations for the prediction of concrete flow without conduction real flow tests. Lowke et al. used CFD simulations for the rheological optimization of flowable concrete [48]. Gram et al. compared the flow time of concrete in an L-box for a defined flow length to viscosity values and validated the model using CFD simulation [49]. Recent investigations implemented time-dependent hydration and flocculation in flow prediction formulas using the discrete element method (DEM), allowing the analysis of the suspension flow on a micro-level implementing particle interactions and boundary conditions [50,51]. Indeed, a comprehensive knowledge of the effect of different rheological parameters on flow behavior, especially for concretes which are less flowable than SCC, sticky, or thixotropic, is missing. Moreover, the rheological input parameters for simulations, especially yield stress as a determining parameter for the analysis or prediction of concrete flow, are sensitively dependent on the way of investigation, which complicates the evolution and application of correct simulation equations. 

## 3. Materials and Methods 

### 3.1. Concept of Investigation

Different rheological investigations on cement pastes and mortars were pursued for the investigation of the correlation of empirical stoppage tests and rheometric measurements as well as to analyze the effect of structural build on flow behavior. Hence, cement pastes with practical relevant actual solid volume fractions of $\phi_{act}$ = 0.45 (0.45_p) and 0.52 (0.52_p) were prepared. For the mortar series, sand from 0 mm to 2 mm was added (0.45_m; 0.52_m). All testing series contained a polycarboxylatether-based superplasticizer (PCE), for which the amount was adjusted to reach mini slump flow values (according to DIN EN 12350-8 [45]) of 200, 225, 250, 275, and 300 ± 5 mm, respectively. The overall concept of the investigation thus includes a variation of viscosity (through different actual solid volume fractions) and yield stress (through different slump flow values), and applies for cement paste and mortar. 

Cement paste series with Ordinary Portland Cement (OPC) CEM I 42.5 R [52] and demineralized water were prepared according to DIN EN 196-1 [53]. The cement was stored at 20 °C without solar irradiation. The mixer was a standard mortar mixer. For cement paste mixtures, a volume of 1.5 L was produced, and for mortar mixtures, a volume of 2.0 L resulted. The investigated mixtures are shown in Table 1.

**Figure 2:**
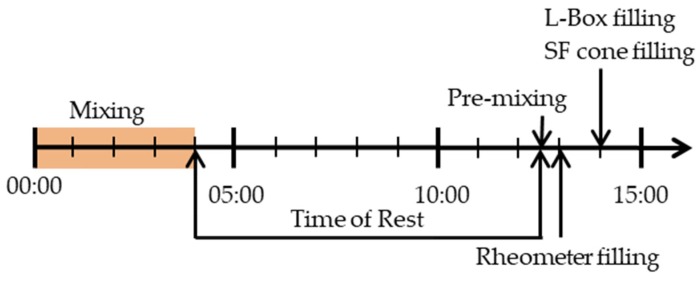
Test procedure from the moment of water addition until the start of rheometric measurements 15:00 min after water addition.

The water was added to the cement immediately before starting the mixing process. For mortar series, cement and sand were mixed homogeneously before the addition of water. After mixing for 90 s at 140 ± 5 rpm, superplasticizer was added during 30 s of rest. Following, the mixture was mixed for another 90 s at 285 ± 10 rpm. Before starting the rheological investigations as well as empirical stoppage tests, the mixture rested from 08:30 min until 12:30 min after water addition. The time of rest was chosen to prevent heterogeneous material behavior from first dissipation which leads to heterogeneous first hydration reactions and therefore slightly different rheological behavior. After the time of rest, the mixture was again mixed for another 30 s. A drilling machine with a propelling screw and a rotational speed of 1700 rpm was taken to ensure homogeneous sample reference states regarding the deflocculation state. At t = 13:00 min after water addition the measurement setups for the rheological as well as the stoppage tests were prepared simultaneously. The mini slump flow was measured 13:30 min after water addition. At t = 15:00 min after water addition, rheometric measurements as well as L-box flow measurements started. The whole procedure can be seen in Figure 2. 

### 3.2. Rheological Measurements 

**Figure 3:**
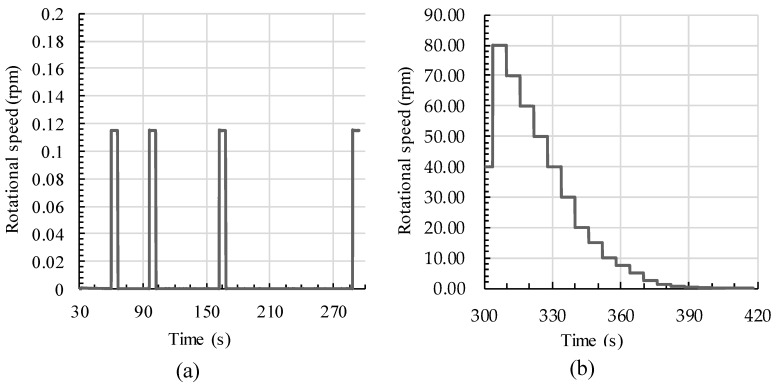
Shear profile for rheological measurements: (**a**) Static profile for thixotropy investigation and (**b**) dynamic profile with a stepwise decreasing rotational speed.

The rheological measurements were conducted using an Anton Paar MCR 502 rheometer (Anton Paar GmbH, Ostfildern, Germany). The geometric device had a vane-in-cup geometry with a six-bladed vane. The cup contained a corrugated surface to prevent wall slip. Directly after 30 s of mixing with the drilling machine, the sample was filled into the vane cup. The rheometric measurements were started with a static shear profile followed by a dynamic one with stepwise decreasing rotational speeds (see Figure 3).

The static profile started with 30 s of preshearing at 80 rpm to break the already built flocs during rheometer preparation (not shown in Figure 3). After the preshearing, a 30-s resting period was conducted followed by an immediate rotational speed of 0.1155 rpm for 6 s. For the calculation of the static yield stress $\tau_{0,s}$, two resting periods of 60 and 120 s were set. During the resting time, the cement paste rebuilt its thixotropic structure; thus, the measured peak torque increased over time. The resulting stress was calculated from each measured peak torque due to geometric conversion. The peak shear stress was subsequently evaluated as static yield stress and the linear increase of static yield stress over the time was calculated as thixotropy $A_{thix}$. After the static measurement, the cement paste was presheared in the vane cup with a rotational speed of 40 rpm for 10 s. Following the preshearing, the dynamic profile was performed with 19 stepwise decreasing rotational speeds from 80 rpm until 0.02 rpm, with a shear time of 6 seconds per step. The average torque was calculated from the equilibrium state for each step, i.e. the last 2 s of each step. The shear stress was calculated from the torque value following geometric basics according to the Reiner–Riwlin equation [39,54]. The yield stress $\tau_{0,B}$ for each flow curve was calculated following the Bingham regression for the steady state between 20 rpm and 60 rpm. The viscosity was taken as plastic viscosity μ for the same flow region. 

### 3.3. Empirical Stoppage Tests

**Figure 4:**
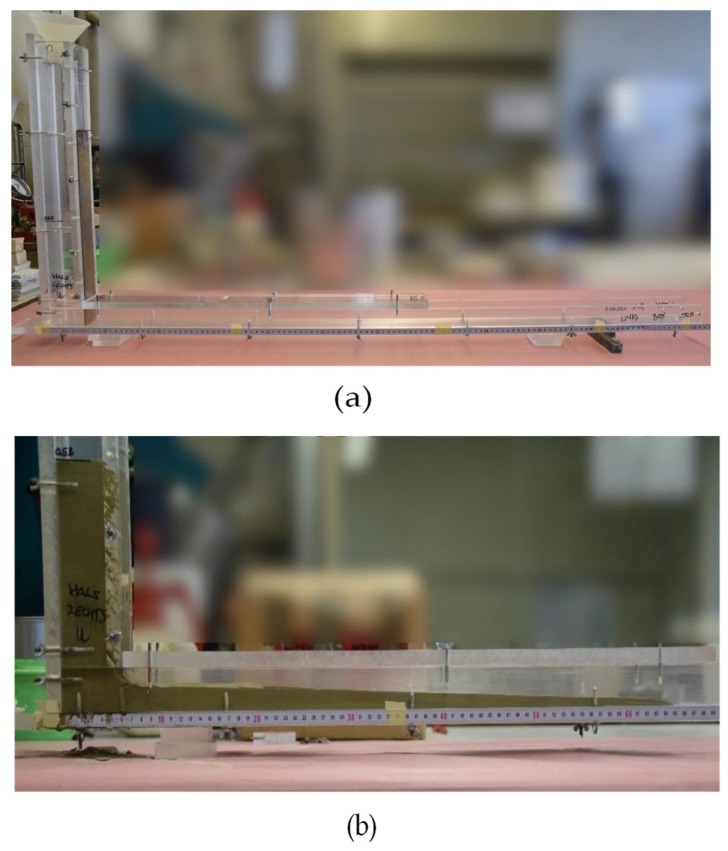
L-box (**a**) before and (**b**) during form-filling of cement paste.

The L-box used for the empirical stoppage tests (compare Figure 4) possessed a modified geometry compared to standardized L-boxes or in comparison to recent research publications. A small L-box was invented for a test volume of 0.5 L. The geometric profile and preliminary investigations are published in [55]. The horizontal section of the L-box has an inner width of 50 mm and a length of 1200 mm with a clearance height of 50 mm. The vertical section exhibits an inner width of 50 mm and an inner depth of 45 mm, with a total height of 540 mm.

Both sections are separated with a gate. For the investigations general assumptions were made: The L-box tests were conducted at a low speed where free flow was assumed. Wall effects and inertia were neglected. In [48] the dam break problem was taken into account causing turbulent flow when lifting the gate. In the presented investigations, due to slow gate lifting, laminar flow was assumed. Following, the sample was driven slightly by hydrodynamic pressure. 

The test procedure (Figure 2) was passed simultaneously to the rheological measurements. After preshearing of 30 s, from 12:30 min to 13:00 min after water addition, 0.5 L of sample amount was filled into the L-box. Before lifting the gate, the sample was left at rest for another 30 seconds. Subsequently, the gate was lifted slowly to avoid inertia effects. The duration for lifting the gate was 5 s, starting at t = 15:00 min after water addition, which ensured a similar testing time for the L-box and the mini slump for testing. The sample was left at flow until flow no longer occurred. The flow distance was measured subsequently. The final height at the gate entrance was considered as h_0_ so the yield stress could be calculated according to Equation (4). The whole flow procedure was video recorded. This enables a flow analysis with flow velocity. The flow velocity was calculated in mm/s. 

## 4. Results and Discussion

### 4.1. Rheological Parameters

**Figure 5:**
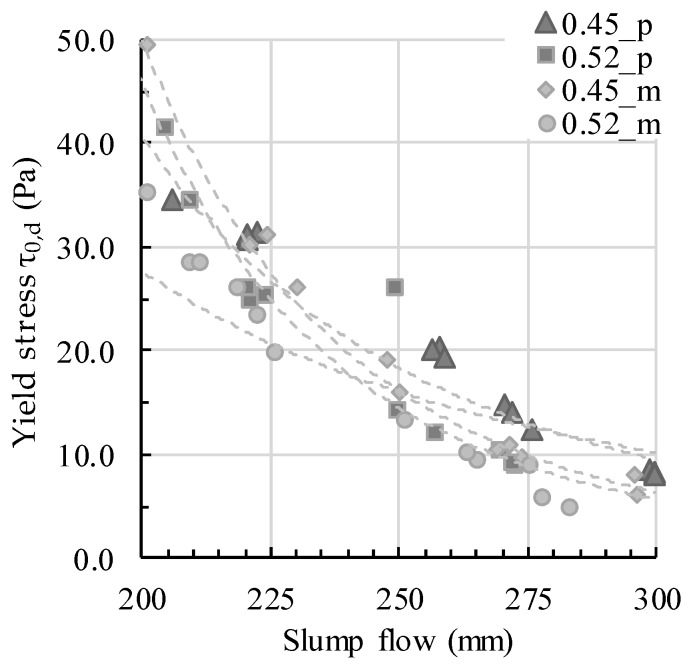
Calculated dynamic yield stress τ_0,d_ depending on the slump flow value.

**Figure 6:**
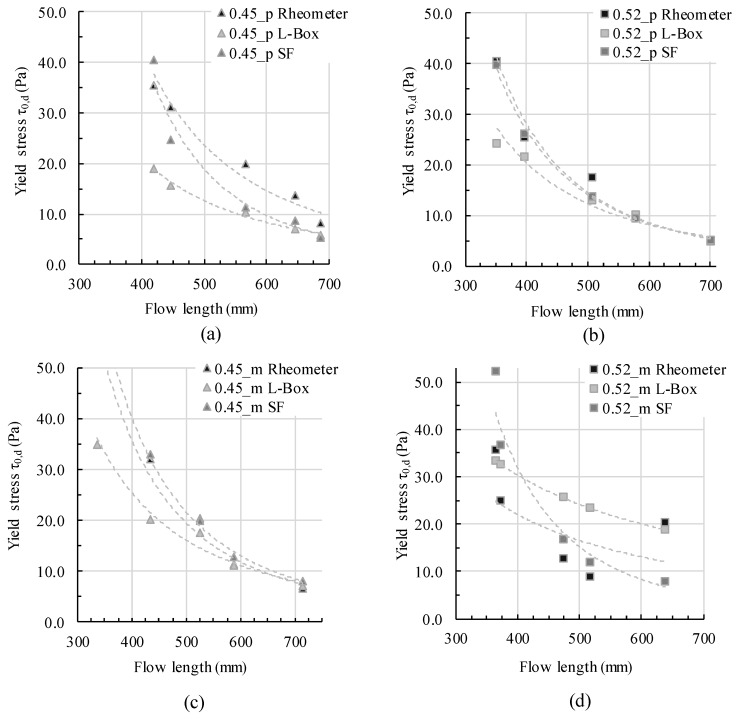
Flow length depending on yield stress, calculated based on rheometer data, L-box results, and slump flow diameter for (**a**) paste 0.45_p; (**b**) paste 0.52_p; (**c**) mortar 0.45_p; and (**d**) mortar 0.52_m.

The rheological parameters of yield stress, viscosity, and thixotropy were calculated as described in Section 3. The calculated parameters are presented in Table 2. All parameters are the average from three individual measurements. The results describe an expected decrease of the calculated Bingham yield stress $\tau_{0,B}$ with an increase of the mini slump flow value, which was calculated with the mini slump flow yield stress $\tau_{0,sF}$ as well, and a decrease in calculated plastic viscosity μ. Also, with increasing slump flow the flow length generally increases, which leads to decreased calculated L-box yield stress $\tau_{0,L-Box}$. Moreover, with increasing PCE amount, which decreases attractive particle interactions, the thixotropy $A_{thix}$ of the tested samples decreased. For a better comprehension and description of measured values and their rheological correlations, Figure 5 and Figure 6 are provided. Roussel et al. assume the correlation between dynamic yield stress and the mini slump flow value [10]. The correlation between the two parameters calculated with Equation (3) is shown in Figure 5 for all testing series. The general assumption that yield stress decreases with increasing slump flow is valid for all series and serves as base for the subsequent comparison of yield stress values in correlation with workability (L-box flow). 

### 4.2. Flow Analysis 

Step 1: Applying yield stress values

In Figure 6, the L-box flow length is compared to the dynamic yield stress from the three aforementioned yield stress calculation approaches: the rheometric investigation $\tau_{0,B}$, calculated yield stress according to the mini slump flow test $\tau_{0,SF}$, and calculated yield stress according to the L-box test $\tau_{0,LBox}.$ Generally, for low flow lengths the calculated yield stress from L-box flow is always the lowest, followed by the yield stress calculated from slump flow value. The measured rheometric yield stress is the highest. With increasing flow length, the gap between calculated and measured yield stresses decreases. For three of the four testing series (0.45_p, 0.45_m and 0.52_p), the yield stresses at the end of flow are very similar. For the last testing series, 0.52_m, yield stress calculated from L-box flow is generally the highest. Subsequently, there is no all-in-all correlation for only yield stress and L-box flow. In particular, the slope and the gap between calculated and measured yield stresses vary depending on the mixture composition. Thus other rheological parameters like viscosity and structural buildup have to be taken into account. 

For the analysis and interpretation, three aspects should be considered:

- The rheometric dynamic yield stress measurement took place after the static yield stress measurement. The high shear of 30 seconds before the dynamic shear profile served for a homogenization and deagglomeration after the time of rest. Still, the agglomerate network within the colloidal paste changed over time and with rest, with a major impact on rheometric values (cf. [38])
- The L-box flow was assumed as free flow. Still, within a short time of rest a low hydrodynamic pressure from the top to the bottom throughout the vertical column built up, which pushed the flow as soon as the gate was lifted.
- The rheometric measurements were conducted with a vane-in-cup system using the Reiner–Riwlin equation for the calculation of shear stress from rotational torque. With increasing non-Bingham-like material behavior, the regression fits less.

The mentioned aspects, whose content leads to poorer comparability of the values, present difficulties in rheometric investigations. For a better understanding of the correlation of different rheological parameters depending on mixture composition and form-filling behavior of cementitious materials, not only yield stress but also viscosity was taken into account in a second step. 

Step 2: Applying velocity values 

**Figure 7:**
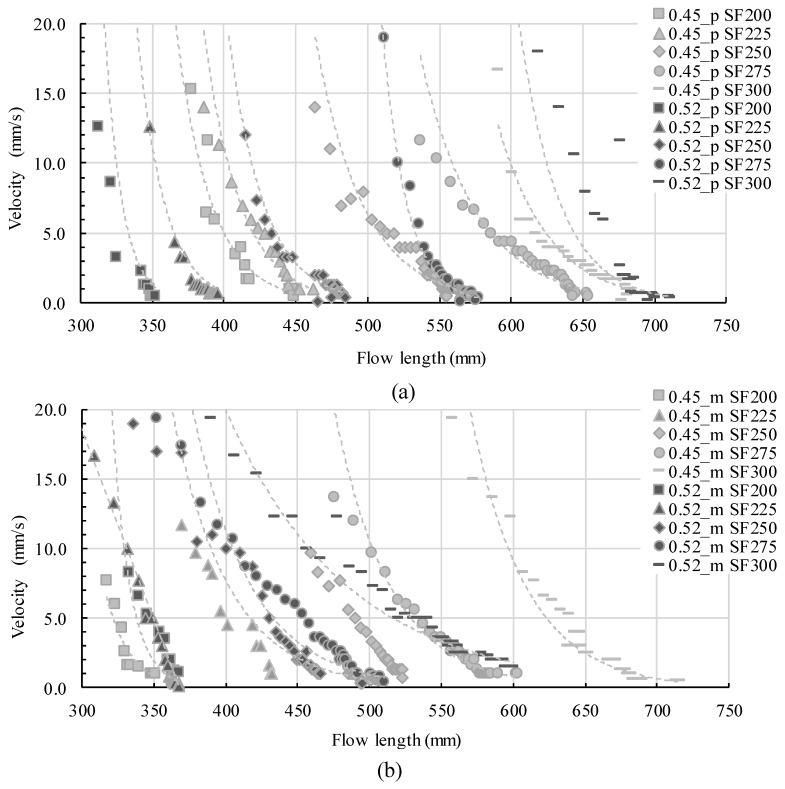
Velocity profiles for (**a**) paste series and (**b**) mortar series.

Cement paste and concrete possess shear-dependent viscosities. Plastic viscosity during steady shear might suffice for relative comparisons or the estimation of flow velocity during steady flow. Indeed, during form-filling the flow velocity decreases until rest. An estimation of shear rate and thus the correlation of a shear rate-dependent viscosity to each part of flow is difficult and needs numerical simulation. Thus, for a simple demonstration, the velocity depending on flow length was calculated for each paste (Figure 7a) and mortar (Figure 7b). The data points are calculated data points for each second of flow during a flow time from two seconds after flow start until the end of flow. The dashed lines show the potential decreasing trend functions.

After the gate is lifted, flow occurs with maximum velocity. The maximum flow velocity is furthermore increased with increasing the slump flow values of the mixtures. Therefore the velocity calculation shown in Figure 7a for all pastes and Figure 7b for all mortars invariably presents flow velocity ongoing from two seconds after gate-lifting. Analyzing the velocity for each mixture, some conclusions can be drawn:

- Within the first two seconds, the flow length is different for each slump flow value. The higher the slump flow value, the higher the flow velocity and thus the initial flow length after two seconds
- The velocity decreases tremendously but with a reduced rate during the experiment
- The change of velocity decrease is more pronounced for highly flowable mixtures and for mortars than for pastes
- Very slow flow takes place for the last few centimeters of flow

Step 3: Applying thixotropy at rest

**Figure 8:**
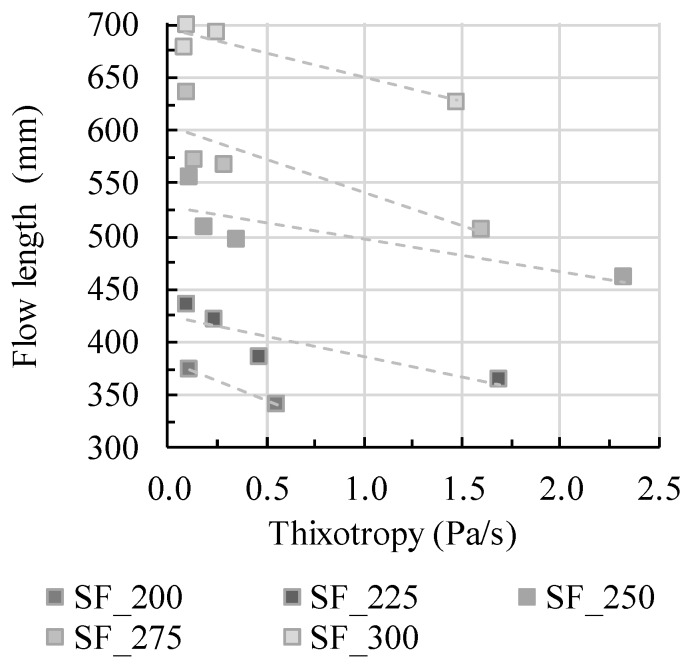
Effect of thixotropy value *A_thix_* on the flow distance depending on yield stress adjustment.

With a change of velocity during flow, rheological parameters change. For shear-thinning mixtures, viscosity increases with decreasing velocity and thus shear rate. Therefore, not only a constant viscosity parameter should be taken into account for the calculation of concrete flow but either a flow-dependent viscosity or just the velocity. Moreover, with decreasing velocity and thus shear rate, structural buildup increases. Therefore, thixotropic structural buildup should be taken into account for flow analysis during form-filling, which was previously shown by the author in [55]. In Figure 8, a general correlation of thixotropy values (while containing the same initial slump flow values and thus dynamic yield stress) and flow length shows the decrease of flow length with increase in thixotropy. In the diagram, all mixtures are shown (0.45_p, 0.45_m, 0.52_p, and 0.52_m). For all samples the slump flow was adjusted through different amounts of superplasticizer; therefore it is not distinguished between different samples in the diagram. The depiction between thixotropy and flow length shall be shown solely exemplarily depending on the slump flow value. It is once more shown that taking only initial dynamic yield stress into account is not sufficient to predict form-filling ability. 

**Figure 9:**
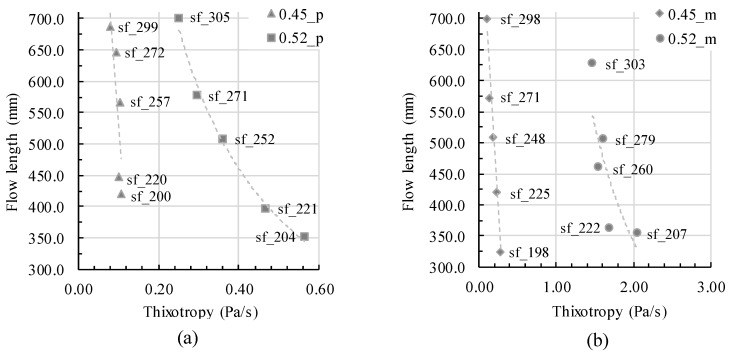
Flow length depending on thixotropy for (**a**) paste series and (**b**) mortar series with an indication of the slump flow value for each sample.

**Figure 10:**
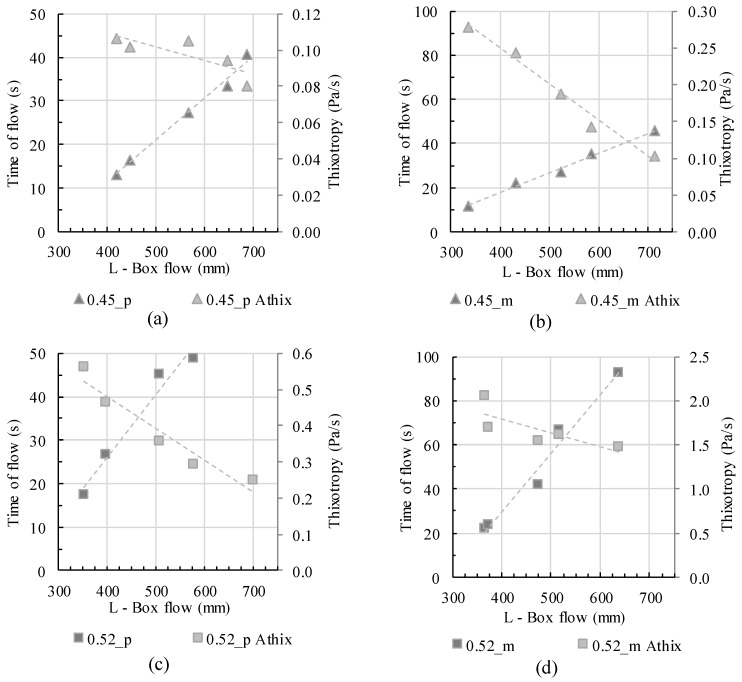
Correlation of L-box flow with flow time and thixotropy.

The testing series 0.45_p and 0.45_m contain a w/c ratio of 0.4, wherefore thixotropic structural buildup due to colloidal forces is thus relatively low for these testing series. The flow length decreases without the impact of thixotropy, which is nearly the same value for all mixtures in the testing series 0.45_p and 0.45_m. The decrease in flow length is pronounced and nearly linear, compare Figure 9. In comparison, the testing series 0.52_p and 0.52_m contain a w/c ratio of 0.3 and thus pronounced thixotropic structural buildup due to lower particle distances and therefore higher interparticle forces. The decrease in flow length correlates to increasing structural buildup; still, the decrease in flow length is less pronounced and shows a nearly potential decrease of flow length (especially Figure 10 a). Obviously, the effect of thixotropy on flow changes. 

Step 4: Combination of rheological parameters 

As shown in steps 1–3, the flow length cannot be depicted by the investigation of only one rheological parameter. Indeed, the flow length is not only dependent on yield stress, viscosity, and structural buildup but on the interaction between these parameters as well. Increasing viscosity implies slower and thus longer flow, which might lead to an increase in thixotropic structural buildup as soon as the velocity gets too low to provide shear rates for full structural breakdown. At the same time, slow flow with subsequent residual structural buildup leads to an increase in static yield stress $\tau_{0,s}$ and therefore even faster stoppage of the flow. The interdependence between these parameters is not trivial and hardly predictable, wherefore a general correlation to common aforementioned equations does not seem appropriate. For a comprehensive depiction of the correlation between the flow time, flow length and thixotropy, Figure 10 is shown. For all mixtures, both the total time of flow until the end of flow and thixotropy are shown in correlation with L-box flow length. 

A general idea of form-filling behavior is not possible. Indeed, different cases of concrete flow can be determined. If no thixotropy occurs, flow length is dependent on yield stress. In Figure 10a the thixotropy of the sample 0.45_p is quite low (0.08–0.11 Pa/s); moreover, the values change only slightly for different flow lengths. It thus can be assumed that the total time of flow and the flow length correlate directly depending on the suspension’s yield stress, as given in Equation (4). The flow predictability is possible without the knowledge of more parameters, and the inertia effects are negligible. The time of flow or the flow velocity do not affect the final flow length. The flow velocity indeed affects the viscosity, which is not constant but dependent on the shear rate: with changing shear rate the viscosity can be shear-thinning or shear-thickening, depending on the relative solid volume fraction. Still, shear-dependent viscosity does not affect the flow length directly as long as no relevant thixotropy occurs. As soon as thixotropy has to be taken into account, the prediction becomes more complicated: the thixotropy of the mortar in Figure 10b is higher and has a wider range (0.25–0.56 Pa/s). The time of flow is not only dependent on yield stress but on thixotropic structural buildup as well. Flow stoppage occurs as soon as thixotropic structural buildup leads a static yield stress which surpasses the suspension’s required yield stress to stop flow. These effects are even more pronounced for the samples in Figure 10c,d. Due to the suspension’s actual solid volume fraction of $\Phi_{act}$ = 0.52, the viscosity generally is already higher than for pastes with $\Phi_{act}$ = 0.45. Due to lower particle distances, higher thixotropy occurs. Slow flow due to higher viscosity and higher residual thixotropy values thus lead to faster flow stoppage, even if the yield stress values (adjusted through the mini slump flow values) theoretically are the same as in the aforementioned series. The calculation of flow length thus is dependent on thixotropy and therefore also on the shear-dependent viscosity. The viscosity determines (1) the speed of flow and thus the shear-dependent effective thixotropic structural build, and (2) the total duration of flow which itself determines the residual time for structural buildup. Therefore, a general flow prediction without knowledge of the interdependencies between the rheological parameters and workability is not easily possible. 

Simulations of concrete flow, e.g., using methods like CFD, can help to investigate the actual shear rate during flow. With the knowledge of the residual thixotropy value per shear rate, an implementation of thixotropy or shear-dependent residual structural buildup will help to predict the actual flow length not only depending on yield stress, but also depending on time of flow and thixotropy. 

## 5. Conclusions

The presented investigations show the form-filling behavior of cement pastes and mortars depending on the rheological parameters of yield stress, viscosity, and thixotropy. The comparability of rheological and workability parameters was studied using common empirical correlation equations. The results allow a sophisticated overview on workability properties from the perspective of form-filling. A general correlation using common equations is not appropriate and does not describe concrete flow properly. Workability always is the sum of viscosity, structural buildup, and initial yield stress. All parameters are also time- and shear rate-dependent, which makes particularly slow flow velocities less predictable. Concretes with low structural buildup and almost Bingham-like material behavior fit to common empirical correlation equations with sufficient accuracy. By increasing the actual solid volume fraction and the structural buildup, yield stress as well as viscosity and thixotropy have to be taken into account for workability prediction. Graphics like Figure 10, where more parameters and their effect on workability are shown, seem to provide a good description for the effect of the aforementioned parameters on workability. 

The results of the experimental program serve as input parameters for more detailed workability formulation equations for the prediction of concrete flow, e.g., simulation in CFD. Nevertheless, a broader experimental program has to be conducted to serve as a database for the simulation of concrete flow for a broad field of modern concretes. Moreover, different parameters affecting flow, such as steel bars, inertia effects, wall pressure, and hydrodynamic effects, were not taken into account. 

Finally, it can be remarked that many parameters affecting concrete rheology are important for predicting flow behavior. Flowability expressed through rheological parameters is not similar to concrete workability; still, a proper combination of these parameters suffices for prospective workability predictions.

## Figures and Tables

**Table 1:** Cement paste and mortar mixtures.

Mixture	w/c Ratio	Solid Concrete Φ [-]	Cement (kg/m^3^)	Water (kg/m^3^) ^1^	Standard Sand (kg/m^3^)	PCE (wt. % by Cement) ^2^
0.45_p	0.40	0.45	1395.1	551.4	-	0.13–0.34
0.45_m	0.40	0.45	1088.2	430.1	1350 ± 5	0.32–0.57
0.52_p	0.30	0.52	1615.2	480.1	-	0.68–1.24
0.52_m	0.30	0.52	1259.9	375.9	1350 ± 5	2.78–5.44

^1^ The water content of the PCE was subtracted from the full water amount, ^2^ The PCE amount depended on the adjustment of required mini slump flow value.

**Table 2:** Experimental results for all testing series.

Average Slump Flow (mm)	0.45_p						
	$\tau_{0,B}(\mathrm{Pa})$	$\tau_{0,L-Box}(\mathrm{Pa})$	$\tau_{0,SF}$ (Pa)	µ (Pas)	$A_{thix}(\mathrm{Pa}/s)$	Flow Length (mm)	Flow Time (s)
200.0	35.50	18.90	40.54	1.60	0.11	419	13.2
220.7	31.10	15.53	24.63	1.16	0.10	447	16.3
257.3	19.93	10.47	11.42	0.75	0.11	567	27.5
272.3	13.70	6.97	8.61	0.82	0.09	647	33.5
299.2	8.23	5.77	5.39	0.66	0.08	687	40.7
Average slump flow (mm)	**0.52_p**						
	$\tau_{0,B}$ (Pa)	$\tau_{0,L-Box}$ (Pa)	$\tau_{0,SF}$ (Pa)	µ (Pas)	$A_{thix}$(Pa/s)	Flow length (mm)	Flow time (s)
203.7	40.60	24.37	39.89	2.04	0.56	352	17.4
221.3	25.43	21.53	26.14	1.55	0.46	396	26.6
251.7	17.50	12.97	13.78	1.35	0.36	508	45.2
270.8	9.57	10.20	9.53	1.15	0.29	578	48.9
305.3	4.83	4.87	**5.24**	0.86	0.25	701	42.5
Average slump flow (mm)	**0.45_m**						
	$\tau_{0,B}$ (Pa)	$\tau_{0,L-Box}$ (Pa)	$\tau_{0,SF}$ (Pa)	µ (Pas)	$A_{thix}$(Pa/s)	Flow length (mm)	Flow time (s)
197.7	49.80	35.03	63.05	2.75	0.28	336	11.6
224.8	29.20	20.37	33.16	2.41	0.24	434	21.8
248.3	18.03	17.60	20.09	1.95	0.19	524	27.2
271.0	10.33	11.17	12.99	2.08	0.14	586	35.3
298.0	6.47	7.33	8.08	1.60	0.10	714	45.7
Average slump flow (mm)	**0.52_m**						
	$\tau_{0,B}$ (Pa)	$\tau_{0,L-Box}$ (Pa)	$\tau_{0,SF}$ (Pa)	µ (Pas)	$A_{thix}$ (Pa/s)	Flow length (mm)	Flow time (s)
207.0	30.80	33.37	52.16	7.52	2.05	364	21.8
222.0	23.20	32.43	36.61	7.13	1.69	374	23.6
252.7	11.03	25.67	16.83	6.78	2.32	474	41.9
278.5	6.53	23.27	11.78	7.37	1.61	516	66.9
303.0	19.90	18.80	7.76	6.97	1.47	638	92.5

## References

[1] Bingham E.C. (1916). An Investigation of the Law of Plastic Flow. Bull. Bur. Stand..

[2] Bentz D.P., Ferraris C.F., Galler M.A., Hansen A.S., Guynn J.M. (2012). Influence of particle size distributions on yield stress and viscosity of cement–fly ash pastes. Cem. Concr. Res..

[3] Larrard F., de Ferraris C.F., Sedran T. (1998). Fresh concrete: A Herschel-Bulkley material. Mater. Struct..

[4] Banfill P.F.G. The Rheology of Fresh Cement and Concrete—A Review. Proceedings of the 11th International Cement Chemistry Congress.

[5] Koehler E.P., Fowler David W. (2003). Summary of Concrete Workability Test Methods.

[6] Roussel N. (2007). Rheology of fresh concrete: From measurements to predictions of casting processes. Mater. Struct..

[7] Murata J. (1984). Flow and deformation of fresh concrete. Mater. Struct..

[8] Fataei S., Secrieru E., Mechtcherine V., Mechtcherine V., Khayat K., Secrieru E. (2020). Influence of Aggregate Volume Fraction on Concrete Pumping Behaviour. Rheology and Processing of Construction Materials.

[9] Kovler K., Roussel N. (2011). Properties of fresh and hardened concrete. Cem. Concr. Res..

[10] Roussel N., Stefani C., Leroy R. (2005). From mini-cone test to Abrams cone test: Measurement of cement-based materials yield stress using slump tests. Cem. Concr. Res..

[11] Nguyen T.L.H., Roussel N., Coussot P. (2006). Correlation between L-box test and rheological parameters of a homogeneous yield stress fluid. Cem. Concr. Res..

[12] DIN EN 12350-10 (2010). Testing Fresh Concrete—Part 10: Self-Compacting Concrete—L Box Test.

[13] Barnes H.A. (1997). Thixotropy—A review. J. Non-Newton. Fluid Mech..

[14] Cheng D.C.-H. (1987). Thixotropy. Int. J. Cosmet. Sci..

[15] Dullaert K., Mewis J. (2005). A model system for thixotropy studies. Rheol. Acta.

[16] Roussel N. (2006). A thixotropy model for fresh fluid concretes: Theory, validation and applications. Cem. Concr. Res..

[17] Glotzbach C., Lowke D., Kränkel T., Mazanec O., Schmidt M., Gehlen C., Schmidt M., Fehling E., Fröhlich S., Thiemicke J. (2014). Anwendungsorientierte Optimierung und Klassifizierung der rheologischen Eigenschaften von UHPC—Optimization and Classification of Rheological Properties of UHPC for its Application. Nachhaltiges Bauen mit Ultrahochfestem Beton—Sustainable Building with Ultra-High Performance Concrete.

[18] Mujumdar A., Beris A.N., Metzner A.B. (2002). Transient phenomena in thixotropic systems. J. Non-Newton. Fluid Mech..

[19] Lowke D., Kränkel T., Gehlen C., Schießl P., Khayat K.H., Feys D. (2010). Effect of Cement on Superplasticizer Adsorption, Yield Stress, Thixotropy and Segregation Resistance. Design, Production and Placement of Self-Consolidating Concrete.

[20] Thrane L.N. (2007). Form Filling with Self-Compacting Concrete. Ph.D.Thesis.

[21] Roussel N., Gram A., Cremonesi M., Ferrara L., Krenzer K., Mechtcherine V., Shyshko S., Skocec J., Spangenberg J., Svec O. (2016). Numerical simulations of concrete flow: A benchmark comparison. Cem. Concr. Res..

[22] Barnes H.A., Hutton J.F., Walters K. (1993). An Introduction to Rheology.

[23] Barnes H.A. (1999). The yield stress—A review or panta rei—Everything flows?. J. Non-Newton. Fluid Mech..

[24] Choi M., Park K., Oh T. (2016). Viscoelastic properties of Fresh Cement Paste to Study the Flow Behavior. Int. J. Concr. Struct. Mater..

[25] Hafid H., Ovarlez G., Toussaint F., Jezequel P.H., Roussel N. (2016). Effect of particle morphological parameters on sand grains packing properties and rheology of model mortars. Cem. Concr. Res..

[26] Qian Y., Kawashima S. (2016). Flow onset of fresh mortars in rheometers: Contribution of paste deflocculation and sand particle migration. Cem. Concr. Res..

[27] Liu D.-M. (2000). Particle packing and rheological property of highly-concentrated ceramic suspensions: Φm determination and viscosity prediction. J. Mater. Sci..

[28] Genovese D.B. (2012). Shear rheology of hard-sphere, dispersed, and aggregated suspensions, and filler-matrix composites. Adv. Colloid Interface Sci..

[29] Da Cruz F., Chevoir F., Bonn D., Coussot P. (2002). Viscosity bifurcation in granular materials, foams, and emulsions. Phys. Rev. E.

[30] Firth B.A., Hunter R.J. (1976). Flow Properties of Coagulated Colloidal Suspensions: Energy Dissipation in the Flow Units. J. Colloid Interface Sci..

[31] Malkin A.Y. (2013). Non-Newtonian viscosity in steady-state shear flows. J. Non-Newton. Fluid Mech..

[32] Yammine J., Chaouche M., Guerinet M., Moranville M., Roussel N. (2008). From ordinary rhelogy concrete to self compacting concrete: A transition between frictional and hydrodynamic interactions. Cem. Concr. Res..

[33] Coussot P., Nguyen Q.D., Huynh H.T., Bonn D. (2002). Viscosity bifurcation in thixotropic, yielding fluids. J. Rheol..

[34] Roussel N., Le Roy R., Coussot P. (2004). Thixotropy modelling at local and macroscopic scales. J. Non-Newton. Fluid Mech..

[35] Thiedeitz M., Kränkel T., Gehlen C. Thixotropic Structural Build-Up of Cement Pastes at Low Shear Rates. Proceedings of the International Conference on Sustainable Materials, Systems and Structures (SMSS2019).

[36] Wallevik O.H., Feys D., Wallevik J.E., Khayat K.H. (2015). Avoiding inaccurate interpretations of rheological measurements for cement-based materials. Cem. Concr. Res..

[37] Thiedeitz M., Kränkel T., Gehlen C. Effect of the mixing time on rheological parameters of cement pastes. Proceedings of the Conference Proceedings Rheologische Messungen an Baustoffen 2019.

[38] Haist M.E.A. (2020). Interlaboratory study on rheological properties of cement pastes and reference substances—Comparability of measurements performed with different rheometers and measurement geometries. Mater. Struct..

[39] Feys D., Cepuritis R., Jacobsen S., Lesage K., Secrieru E., Yahia A. (2017). Measuring Rheological Properties of Cement Pastes: Most common Techniques, Procedures and Challenges. Rilem. Tech. Lett..

[40] Mechtcherine V., Gram A., Krenzer K., Schwabe J.-H., Shyshko S., Roussel N. (2014). Simulation of fresh concrete flow using Discrete Element Method (DEM): Theory and applications. Mater. Struct..

[41] Flatt R.J., Bowen P. (2007). Yield Stress of Multimodal Powder Suspensions: An Extension of the YODEL (Yield Stress mODEL). J. Am. Ceram. Soc..

[42] Ukrainczyk N., Caggiano A., Schicchi D.S., Gilka-Bötzow A., Koenders E., Mechtcherine V., Khayat K., Secrieru E. (2020). Hydrating Cement Particle Interaction Model for Yield Stress Analysis. Rheology and Processing of Construction Materials.

[43] DIN EN 1015-3:2007-05 (2007). Methods of Test for Mortar for Masonry—Part 3: Determination of Consistence of Fresh Mortar (by Flow Table).

[44] DIN EN 12350-8:2010-12 (2010). Testing Fresh Concrete—Part 8: Self-Compacting Concrete—Slump-Flow Test.

[45] Roussel N. (2007). The LCPC BOX: A cheap and simple technique for yield stress measurements of SCC. Mater. Struct..

[46] Chaparian E., Nasouri B. (2018). L-box—A tool for measuring the “yield stress”: A theoretical study. Phys. Fluids.

[47] Shan Z., Yu Z., Shi J. (2015). Experimental investigation of flow of fresh self-compacting concrete in improved L-box. Constr. Build. Mater..

[48] Lowke D., Illguth S., Kränkel T., Gehlen C., Roussel N., Bessaies-Bey H. Rheological Optimization of Flowable Concretes Based on Computational Fluid Dynamics (CFD). Proceedings of the 7th RILEM International Conference on Self-Compacting Concrete and of the 1st RILEM International Conference on Rheology and Processing of Construction Materials.

[49] Gram A., Silfwerbrand J., Lagerblad B. (2014). Obtaining rheological parameters from flow test—Analytical, computational and lab test approach. Cem. Concr. Res..

[50] Li Z., Cao G., Tan Y. (2016). Prediction of time-dependent flow behaviors of fresh concrete. Constr. Build. Mater..

[51] Tan Y., Cao G., Zhang H., Wang J., Deng R., Xiao X., Wu B. (2015). Study on the Thixotropy of the Fresh Concrete Using DEM. Procedia Eng..

[52] Lu Z., Haist M., Ivanov D., Jakob C., Jansen D., Leinitz S., Link J., Mechtcherine V., Neubauer J., Plank J. (2019). Characterization data of reference cement CEM I 42.5 R used for Priority Program DFG SPP 2005 “Opus Fluidum Futurum—Rheology of reactive, multiscale, multiphase construction materials”. Data Brief.

[53] DIN EN 196-1:2016-11 (2016). Methods of Testing Cement—Part 1: Determination of Strength.

[54] Eslami Pirharati M., Ivanov D., Krauss H.-W., Schilde C., Lowke D., Mechtcherine V., Khayat K., Secrieru E. (2020). Numerical Simulation of the Flow Behavior of Newtonian Fluids in a Wide Gap Rheometer by CFD. Rheology and Processing of Construction Materials.

[55] Thiedeitz M., Kränkel T., Gehlen C., Mechtcherine V., Khayat K., Secrieru E. (2020). Thixotropy-Dependent Form Filling Ability of Cement Paste. Rheology and Processing of Construction Materials.

